# Dr. Thos. W. Evans

**Published:** 1869-09

**Authors:** 


*Dr. Thos. W. Evans*, Court Dentist to the Emperor of France, Prince of Wales, etc., paid a short visit to this country in July last. We had a very agreeable visit from the Doctor during his short stay, and found him to be, what we have ever regarded him, a very pleasant gentleman; and one much more youthful in appearance than we had imagined, his name having been familiar to the profession for so long a time.

